# BUILDING COMMUNITY IN PRACTICE AND RESEARCH OF HEALTHY LONG-TERM CARE ENVIRONMENTS

**DOI:** 10.1093/geroni/igae098.1298

**Published:** 2024-12-31

**Authors:** Peggy Chi, Sarah Hunter, Whitney Berta

**Affiliations:** University of Toronto, Toronto, Ontario, Canada; University of Toronto, Toronto, Ontario, Canada; University of Toronto, Toronto, Ontario, Canada

## Abstract

This presentation will describe an initiative based at the Institute of Health Policy, Management and Evaluation in Toronto, Canada, that aims to dismantle silos and build a community in the practice and research of aging environments. The goals and objectives are to share, synthesize, advance, and mobilize knowledge on long-term care homes—the influence of living environments on older adults and working environments on workers and their work. Three knowledge mobilization activities are the means by which our aims are achieved: 1) A series of seminars are curated for experts to disseminate knowledge to a broad audience (industry and academic leaders, decision-makers, and end-users) to connect research to practice. 2) Informal, practical discussions are created for practitioners to share current key challenges in long-term care homes with researchers to connect practice to research. 3) A Design Charrette is organized to translate knowledge to address key challenges within interdisciplinary teams comprising practitioners/ care providers, team leads, and trainees. These activities bring together individuals from industry and academia who represent diverse disciplines that have generated knowledge about these linkages based on different and complementary conceptualizations of long-term care environments but for whom there is no pre-existing forum in which to engage in integrative, synthetic discussion and application. The open-forum nature of the activities is intended to foment public discourse on the role of physical and psychosocial environments in the health of older adults and workers in long-term care homes and to demonstrate the value of evidence-based design founded on multidisciplinary knowledge.

